# Genetic variability of the core protein in hepatitis C virus genotype 4 in Saudi Arabian patients and its implication on pegylated interferon and ribavirin therapy

**DOI:** 10.1186/1479-5876-12-91

**Published:** 2014-04-06

**Authors:** Fatimah S Alhamlan, Mohammed N Al-Ahdal, Nisreen Z Khalaf, Ayman A Abdo, Faisal M Sanai, Hamad I Al-Ashgar, Mahmoud ElHefnawi, Amina Zaid, Ahmed A Al-Qahtani

**Affiliations:** 1 Department of Infection and Immunity, King Faisal Specialist Hospital and Research Center MBC 03, P.O. Box 3354, Riyadh 11211, Saudi Arabia; 2 Department of Pathology and Laboratory Medicine, King Faisal Specialist Hospital and Research Center, Riyadh, Saudi Arabia; 3 Department of Microbiology and Immunology, College of Medicine, Alfaisal University, Riyadh, Saudi Arabia; 4 Liver Disease Research Center, King Saud University, Riyadh, Saudi Arabia; 5 Department of Medicine, Section of Gastroenterology, College of Medicine, King Saud University, Riyadh, Saudi Arabia; 6 Hepatobiliary Sciences and Liver Transplantation, King Abdulaziz Medical City, Riyadh, Saudi Arabia; 7 Department of Medicine, King Faisal Specialist Hospital & Research Center, Riyadh, Saudi Arabia; 8 Biomedical Informatics and Chemo Informatics group, Informatics and Systems Dept., National Research Centre, Cairo, Egypt; 9 Scientific Institute and Research Academy (SIRA-Corp), Cairo, Egypt; 10 Molecular Diagnostics and Therapeutics Department, Genetic Engineering and Biotechnology Research Institute, Sadat City University, Monofia, Egypt

**Keywords:** Hepatitis C Virus (HCV) Treatment, Genotype 4, PEG-IFN/RBV combined therapy

## Abstract

**Background:**

Hepatitis C virus (HCV) shows a remarkable genetic diversity, contributing to its high persistence and varied susceptibilities to antiviral treatment. Previous studies have reported that the substitution of amino acids in the HCV subgenotype 1b core protein in infected patients is associated with a poor response to pegylated interferon and ribavirin (PEG-IFN/RBV) combined therapy.

**Objectives:**

Because the role of the core protein in HCV genotype 4 infections is unclear, we aimed in this study to compare the full-length core protein sequences of HCV genotype 4 between Saudi patients who responded (SVR) and did not respond (non-SVR) to PEG-IFN/RBV therapy.

**Study design:**

Direct sequencing of the full-length core protein and bioinformatics sequence analysis were utilized.

**Results:**

Our data revealed that there is a significant association between core protein mutations, particularly at position 70 (Arg^70^Gln), and treatment outcome in HCV subgenotype 4d patients. However, HCV subgenotype 4a showed no significant association between core protein mutations and treatment outcome. In addition, amino acid residue at position 91 was well-conserved among studied patients where Cys^91^ is the dominant amino acid residue.

**Conclusions:**

These findings provide a new insight into HCV genotype 4 among affected Saudi population where the knowledge of HCV core gene polymorphisms is inadequate.

## Background

Hepatitis C virus (HCV) infects more than 170 million people worldwide leading to chronic hepatitis, cirrhosis and hepatocellular carcinoma [1]. HCV belongs to the family *Flaviviridae* and is a member of hepacivirus genus. It is classified into seven genotypes and numerous subtypes [2,3]. HCV has a single-stranded RNA that encodes a polyprotein which subsequently gets cleaved into number of structural and non-structural proteins. Although the function of each protein has been intensively studied, the point mutations that occur in various positions and cause antiviral drug resistance are largely unknown. Therefore, the study of variation at the nucleotide sequence of HCV, core protein in particular, from different geographical region is important to understand its prevalence in the world as well as its clinical management.

Recently, advances in HCV treatment have led to the development of many direct-acting antiviral (DAA) agents. Early this year, the U.S. Food and Drug Administration (FDA) has approved a new therapy (simeprevir) to treat chronic HCV infection [4]. However, the standard treatment for chronic hepatitis C infection in the developing countries is pegylated interferon (PEG-IFN) plus ribavirin (RBV) where the expected outcome of the treatment is to attain a sustained virological response (SVR) [5]. There are serious side-effects and high medical cost that are associated with PEG-IFN/RBV treatment. As a result, it is important to predict the response to therapy for each individual patient beforehand. Previous studies have shown that the sequence polymorphisms within viral proteins, such as core protein, correlate with IFN-based treatment outcome. For example, substitutions of amino acid 70 and/or 91 in HCV subgenotype 1b core protein are predictors of poor response to PEG-IFN/RBV treatment [6,7]. The clinical advantage of predicting SVR to PEG-IFN/RBV in patients is that patients with Arg^70^/Lue^91^ residues ought to continue the treatment course with predicted positive response. However, in patients who have mutated residues in the core region (Gln^70^/Met^91^) would be advised to withdraw from the treatment to avoid unnecessary side-effects. Indeed, if a correlation between HCV core gene mutation(s) and treatment outcome is established, then HCV sequencing can become a noninvasive and economical tool to assess an individual status and response to a treatment.

Although HCV genotype 4 is the cause of approximately 20% of HCV infection worldwide, it is poorly studied [8]. Furthermore, there are limited studies and low informative data from patients in Saudi Arabia who are infected with HCV genotype 4. The aim of this study is to analyze the core protein of HCV genotype 4 from Saudi patient isolates and investigate the association between core protein sequence variations and treatment outcome.

## Methods

### Study patients and treatment regimens

The study protocol was approved by the local ethics committee at King Faisal Specialist and Research Center and written informed consent was obtained from each patient. A total of 115 baseline (i.e., treatment-naïve) patients from three different hospitals (King Khalid University Hospital, King Faisal Specialist Hospital and Research Center, and Riyadh Military Hospital) in Riyadh, Saudi Arabia, were used in this study. Exclusion criteria included co-infection with hepatitis B or human immunodeficiency virus, co-existent autoimmune or metabolic liver disease, active drug-induced hepatitis, decompensated cirrhosis, evidence of severe retinopathy, neoplastic disease, coronary artery or cerebrovascular disease, history of clinically relevant psychiatric disease. The complete treatment protocol used for these patients was previously published [9]. HCV RNA extraction, genotyping and subgenotyping were determined using previously described methods [10]. Herein, we presented the most dominant subgenotypes of HCV genotype 4 that are HCV-4d and HCV-4a in each group (SVR and non-SVR). Due to limited sample size, we excluded 4r, 4n and 4o from data analysis.

### HCV sequence alignment and primer design

Complete genome sequences of HCV from different geographical regions were retrieved from the GenBank database (http://blast.ncbi.nlm.nih.gov/Blast.cgi). Multiple sequence alignment of the retrieved sequences was performed using ClustalW module of MegAlign software (DNASTAR, Inc.,) and the consensus sequence was used to design degenerate primers for the core region. Primer sequences and positions are as follows: Forward: 5' TGCTAGCCGAGTAGTGTTGG 3' (positions 246–268) Reverse: 5' CCARTTCATCATCATRTCCCA 3' (position 1298–1318) and the amplicon size is 1045 bp.

### Polymerase chain reaction (PCR)

All PCR mixtures had a total volume of 25 μl that contained 1 μl of HCV cDNA, 12.5 μl of GoTaq® Green Master Mix (Promega, Madison, USA), 1 μM of forward and reverse primers, and sterile nuclease-free water. In addition, appropriate positive and negative controls were employed. PCR conditions were as follows: 2 min an initial denaturing step at 95°C, followed by 35 cycles of 30 sec denaturing step at 95°C, 1 min of annealing step at 56°C, and 1 min of extending step at 72°C. A final extension at 72°C for 5 min was performed. PCR amplicons were visualized on a 1.5% agarose gel and stained with ethidium bromide. The positive amplicons were processed further for PCR sequencing using ABI3730XL sequencer (Applied Biosystems, Foster City, CA). To confirm positive results, nucleotide sequences were blasted against NCBI database.

### Data analysis and statistics

Sequence chromatograms of 115 full-length core gene sequences were aligned and edited using the Lasergene suite for sequence analysis (DNASTAR, Inc.,) [11]. Nucleotide (573 bp) and amino acid (191 aa) sequences from different patient isolates were aligned using ClustalX module (MegAlign, DNASTAR, Inc.). Full-length core gene sequences of HCV genotype 4 were retrieved from GenBank and used in this study as references. BioEdit program was used to visually display the full-length core protein with genotype corresponding references [12]. In addition, phylogenetic tree was constructed using HCV genotype 4 patient sequences (all subgenotypes were included) and 20 random sequence references. The neighbor-joining method with a bootstrap value of 1,000 replications was employed in constructing the tree using Mega 5.0 software [13].

Further, detecting the most statistically significant differences between the responders and non-responders groups was done using the Viral Epidemiology Signature Pattern Analysis (VESPA) tool, provided by HCV sequence database [14]. Numerical data were analyzed by Student’s *t* test using STATA IC/13 software (StataCorpLP, Houston, USA) where a *P* value of <0.05 was considered statistically significant.

## Results

### Response to PEG-IFN/RBV therapy

One hundred and fifteen (115) patients with chronic HCV genotype 4 were enrolled in this study. The patients’ clinical characteristics are presented in Table 1. Notably, there was no significant association between the response to treatment and age, gender, weight, liver enzymes, HCV viral load, disease stage, and grade. However, there is a significant association between subgenotypes and treatment response. Indeed, SVR rate in HCV-4a is 58% while the SVR rate in HCV-4d is lower (35%) (*P* value = 0.02). Twenty four weeks after the completion of 48 weeks of PEG-IFN/RBV combined treatment; patients were tested and then divided to responders (i.e., SVR (48%)) and non-responders (i.e., non-SVR (51%)). HCV genotype 4 mean genetic distance was calculated between SVR and non-SVR patients (Table 2). All reported sequences in this study were deposited in the GenBank and were assigned the following accession numbers (KC143812 - KC143908).

**Table 1:** Patient characteristics of all patients enrolled in this study

**Variable**	**R**	**NR**	**P-value**
**Age (yrs.)**			
Mean ± SD*	45.82 ± 14.67	49.19 ± 15.26	0.152^c^
Median (25th-75th)^¶^	48.00(35.50-57.00)	52.50(44.50-59.00)	0.095^a^
**Sex**^§^			
Male count (%)	21(37.5%)	22(37.3%)	0.862^b^
Female count (%)	35(62.5%)	37(62.7%)	
**Weight (**kg)^*^	74.54 ± 28.01	76.04 ± 18.81	0.609^c^
**Bil** (mg/dL)^*^	10.54 ± 4.89	12.09 ±6.83	0.42^c^
**ALT** (IU/L)^*^	76.59 ±57.5	82.2 ± 76.2	0.424^c^
**AST** (IU/L)^*^	59.27 ± 43.37	53.63 ± 55.81	0.14^c^
**ALP** (IU/L)^*^	98.4 ± 67.8	116.13 ± 60.98	0.538^c^
**AFP** (ng/mL)^*^	8.22 ±17.12	7.76 ± 18.18.47	0.904^c^
**HCV load** log10^**¶**^	5.74(5.21-6.40)	5.88(5.15-6.48)	0.837^a^
Median (25th-75th)^¶^			
**Stage**^**§**^			
≤2 count (%)	24(77.4%)	21(63.6%)	0.227^b^
≥3 count (%)	7(22.6%)	12(36.4%)	
**Grade**^§^			
≥1 count (%)	8(25%)	8(22.2%)	0.788^b^
3 count (%)	24(75%)	28(77.8%)	
**Genotypes**^§^			
4a (%)	39(58%)	28(42%)	0.02^b^
4d (%)	17(35%)	31(65%)	

Bil, Bilirubin; ALT, alanine aminotransferase; AST, aspartate aminotransferase; ALP, alkaline phosphatase, AFP, α-fetoprotein.

*Values are expressed as Mean±SD, ^¶^values are expressed as median (interquartile range), ^§^count (%).

^a:^Nonparametric test (Mann–Whitney U test), ^b:^Chi-squared test, ^c:^independent t-test.

**Table 2:** Summary of sequence analyses of HCV-4 and mean genetic distance

**Genotype**	**Response**	**No. of sequence**	**No. of ref seq**	**Mean genetic distance within groups**	**Mean genetic distance between groups**
**4a**	SVR*	39	1	0.0221	0.027
	Non-SVR	28	1	0.0225	
**4d**	SVR	17	1	0.0173	0.016
	Non-SVR	31	1	0.0133	

*Sustained Virological Response.

Mean genetic distances are calculated using MEGA 5.1 software.

### Phylogenetic analysis of SVR and non-SVR patients

**Figure 1:**
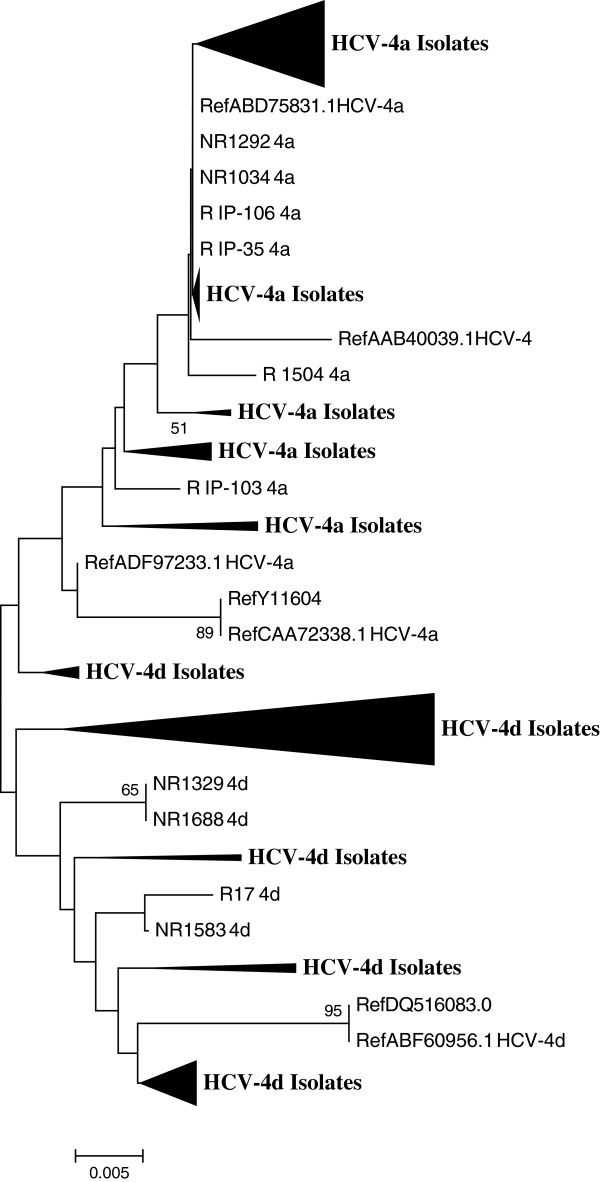
**Phylogenetic tree constructed from sequences of the HCV-4 core protein using neighbor-joining algorithm method.** Bootstrap values corresponding to 1000 replications are indicated below the branches and only bootstrap values > 50% were included. Elongated triangles represent the compressed subtrees with captions indicating HCV isolates and references. The thickness of the triangles is proportional to the number of taxa included and the length of the triangle corresponds to the respective diversity.

Phylogenetic analysis of core sequences provides information about overall relatedness of core gene among HCV genotype 4 isolates. One hundred fifteen sequences of HCV-4 core gene from SVR and non-SVR groups were used to construct the tree (Figure 1). HCV-4 sequences showed no clustering based on response to the treatment but rather they clustered to the respective subgenotypes correctly (i.e. HCV-4a and -4d).

### Multiple sequence alignment of the core protein

**Figure 2:**
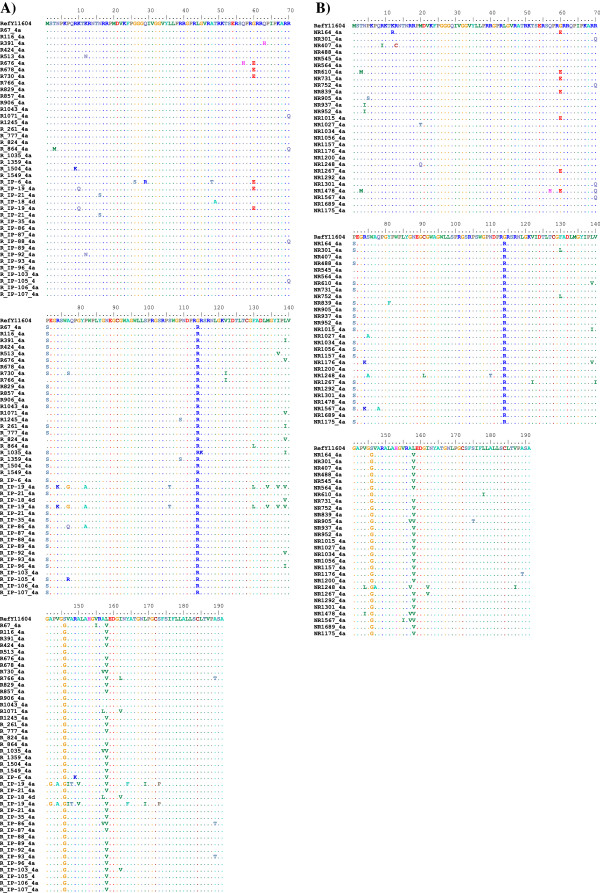
**Amino acid alignment of the core protein of HCV-4a patient isolates. A)** Represents SVR patient amino acid sequences. **B)** Represents non-SVR patient amino acid sequences. Amino acid positions are indicated in the upper part of the figure (i.e. reference sequence). Dots indicate amino acid residues that are similar to the reference sequence while the single-letter code shows amino acid substitutions.

**Figure 3:**
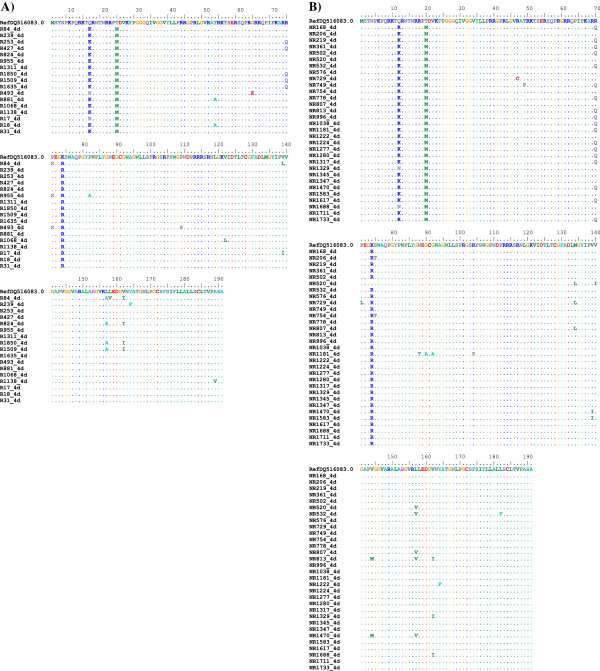
**Amino acid alignment of the core protein of HCV-4d patient isolates. A)** Represents SVR patient amino acid sequences. **B)** Represents non-SVR patient amino acid sequences. Amino acid positions are indicated in the upper part of the figure (i.e. reference sequence). Dots indicate amino acid residues that are similar to the reference sequence while the single-letter code shows amino acid substitutions.

Figures 2 and 3 showed aligned amino acid residues from each group/subgenotype. Figure 2A represents HCV-4a in SVR patients and revealed that the residue at position 70 (Arg^70^) and 91 (Cys^91^) are well conserved among these isolates. On the other hand, our data revealed different point mutations in the core region of HCV-4a in SVR group. At position 60, for example, G (Gly^60^) mutated to E (Glu^60^) in 12% of the clinical samples (Figure 2A). Position 71 also has S (Ser^71^) in 80% of the samples. Position 158 showed substitution of L (Lue^158^) to V (Val^158^) in 82% of the samples. In HCV subgenotype 4a non-SVR patients, however, the amino acid alignment figure revealed that position 70 (Arg^70^) is mutated to (Gln^70^) in only 18.5% of the clinical samples. However, position 71 showed a higher mutation rate were 70% of the clinical samples have S (Ser^71^) instead of P (Pro^71^). Position 60 showed another point mutation that was calculated to be 26% of the clinical samples where E (Glu^60^) substitutes G (Gly^60^). There was no significant correlation between core protein sequences and treatment outcome. Notably, our patient sequences showed a 100% mismatch with the reference sequence in positions such as 114 (G^114^R) and 146 (S^146^G) in HCV-4a (Figure 2).

Figure 3A showed HCV subgenotype 4d in SVR patients where the amino acid alignment revealed that the residue at position 70 (Arg^70^) is mutated to (Gln^70^) in 29% of the clinical samples. Position 71 has a point mutation where P (Pro^71^) is substituted with S (Ser^71^) in only 2% while position 157 has a mutation of L (Leu^157^) to A (Ala^157^) in 23% of the clinical samples. Moreover, position 162 has mutation of V (Val^162^) to I (Iso^162^) in 23%. In non-SVR patients, however, 58% of the clinical samples have mutation at position 70 whereas (Arg^70^) is mutated to (Gln^70^). Moreover, at position 157, 29% of the clinical samples showed mutation of L (Leu^157^) to V (Val^157^), however, this amino acid substitution is different than the mutated amino acid in SVR group (A *vs.* V). There was a significant correlation between HCV-4d core protein sequence at position 70 and treatment outcome (*P* value = 0.02). Moreover, our patient sequences showed a 100% mismatch with the reference sequence in positions such as 12 (Q^12^K), 20 (T^20^M) and 74 (K^74^R) in HCV-4d (Figure 3).

### Patterns discovery and recognition

**Figure 4:**
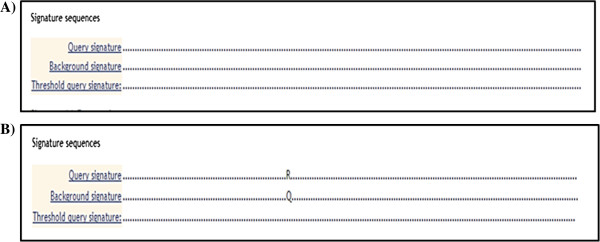
**Viral Epidemiology Signature Pattern Analysis (VESPA).** The query sequence is the SVR group while the background sequence is the non-SVR group. The dots (.) in the signatures indicate that the two sequences agree in those positions while the letter indicates a statistically significant position. **A)** In HCV subgenotype 4a, no signature pattern between the two groups of responders and non-responders was found. **B)** HCV subgenotype 4d results revealed one signature pattern between the query signature (SVR) and Background signature (non-SVR) in genotype 4d. This signature is at position 70, where it is associated with amino acid Arginine (R) in SVR group compared to amino acid Glutamine (Q) in non-SVR group. Statistically significant variations were calculated with a threshold of *P* < 0.05.

Positional variations of the core protein were compared using Viral Epidemiology Signature Pattern Analysis (VESPA). Results revealed that the variations in HCV 4a SVR and non-SVR patients are not statistically significant (Figure 4A), while, the signature pattern analysis of HCV 4d SVR and non-SVR was statistically significant at position 70 (Arg^70^Gln) (*P* value < 0.05) (Figure 4B).

## Discussion

The HCV core gene is the genetic region that encodes for the viral nucleocapsid protein. It consists of 191 amino acid residues that are divided into three domains, an N-terminal hydrophilic domain (D1, residues 1–117), a C-terminal hydrophobic domain (D2, residues 118–170), and the last 21 amino acids that serve as signal peptide for the downstream envelope protein E1 [15,16]. It has been shown that the core protein is associated with number of cellular proteins and pathways that have direct effect on HCV lifecycle and biology [17]. Also, HCV core protein has been suggested to have a role on antiviral activity of IFN inhibition through interaction with the cellular protein, STAT1 [18]. Therefore, mutations in this protein have the potential to alter the viral structure leading to unexpected functions such as poor response to PEG-IFN/RBV therapy. Previous studies have shown that there is a significant correlation between mutations in the core protein and poor treatment outcome. In particular, patients who had substitutions of Arg^70^ to Gln^70^ and/or Leu^91^ to Met^91^ showed lower response to PEG-IFN/RBV combined therapy [19,20]. However, most of these studies have been conducted on Asian populations, especially Japanese patients, who were diagnosed with HCV genotype 1b. Herein, we hypothesized that the amino acid substitutions in HCV genotype 4 (subgenotypes 4a and 4d) core region could correlate with treatment outcome. HCV subgenotype 4d showed that there is a significant association between core protein mutations, particularly at position 70 (Arg^70^Gln), and treatment outcome. However, amino acid substitutions in HCV-4a showed no associations with treatment outcome. The residue at position 70 of the core protein was Arg^70^ in most of HCV-4a SVR patient isolates and only 17% of HCV-4a non-SVR patient isolates were mutated to Gln^70^. Moreover, the residue at position 91 of the core protein was well-conserved among HCV genotype 4.

There are several factors (predictors) that could control the effectiveness of the treatment and such factors can be classified into host and/or viral factors. Host factors include age, gender, patient body weight, ethnicity, alcohol consumption and host genetic variations. Several recent studies have shown that single nucleotide polymorphisms (SNPs) in IL-28B gene region are associated with response to combination therapy with pegylated IFN-α and ribavirin [21]. On the other hand, virus genotypes and viral load have been shown to modulate treatment outcome [6,22]. Based on previous studies, HCV genotype is the most significant factor affecting treatment responses [23]. While HCV genotype 2 and 3 have the highest rate of SVR to PEG-IFN/RBV treatment (80%), HCV genotype 1 and 4 are showing more resistance to treatment (50-60%) [24,25]. Notably, the present study revealed that the SVR rate in HCV-4a is higher (58%) than HCV-4d (35%) indicating a role of the subgenotyping in treatment response. The differences in responding to the treatment among different genotypes and subgenotypes suggest a role of the viral sequence variations. It is noteworthy that most previous studies were conducted on Asian population. Thus, further investigations are needed to explore this phenomenon in different ethnic populations.

In recent studies, El-Shamy *et al.* has investigated 43 Egyptian patients who were infected with HCV genotype 4 (mostly subgenotype 4a) and revealed that no significant correlation between core protein amino acid substitutions at position 70 and/or 91 and treatment outcome [26]. Our finding in regard to HCV-4a is in agreement with the aforementioned report that the substitutions at positions 70 and/or 91 are not associated with antiviral resistance. However, in HCV-4d patient isolates, our data revealed that there is a significant association between core amino acid substitutions, particularly at position 70 and treatment outcome. Phylogenetic analysis and sequence comparison showed that no clustering was observed based on treatment response but rather they grouped to the corresponding subgenotypes correctly (i.e. HCV-4a, −4d).

## Conclusions

The present study revealed that HCV-4d has a point mutation at position 70 (Arg^70^Gln) that is statistically significant. However, no evidence was found in HCV-4a for the effect of core protein polymorphisms, either at position 70 and/or 91, and treatment outcome. Instead, mutations were scattered over the full-length core region with no specific association with drug resistance. Although several possibilities have been proposed to explain the effect of amino acid substitutions of core protein on treatment outcome, the exact mechanism has not been determined. Nonetheless, this study emphasizes the fact that single nucleotide mutations in the core gene could prove helpful in predicting the treatment outcome, at least in sub-genotype 4d-infcted patients.

## Competing interests

The authors declare that they have no competing interests.

## Authors’ contributions

FA carried out the molecular techniques and wrote the manuscript. MA conceived and designed the study. NK carried out the gene sequencing and sequence alignments. AA, FS, HA has contributed substantially by providing the patient specimens and clinical data analysis. ME and AZ have contributed in the bioinformatics analysis. AA conceived and designed this study, interpreted the data, and edited the manuscript. All authors have read and approved the final manuscript.
